# Medical Items and News of Interest to the Physician

**Published:** 1882-04

**Authors:** 


### For the Use of Physicians.

CULLED FROM THE STANDARD MEDICAL JOURNALS OF THE WORLD.

NOTE.—These formulæ are intended only for the regular practitioner of medicine, and should be employed only by him, or under his immediate supervision.

**Quinia as an Antiseptic.**

Dr. Hugo Erichsen, of Detroit, Mich., recommends a solution of quinia as an antiseptic spray, and to apply to wounds in place of carbolic acid solutions.

**Syphilis.**

The use of mercury by hypodermic injections by Dr. Yourn under the following formula has done good service :

℞

Iodide of mercury, 1 gramme.
Iodide of potass, 1 "
Phosphate of soda, tribasic, 2 grammes.
Distilled water, 50 centim. cubic.

This solution is not coagulated by albumen. Mathles has made 850 injections without accident, twenty-five or thirty being sufficient to restore health.

**Cholera Infantum.**

In the Hospital for Infants at Breslau, in 91 cases treated by resorcin, the vomitings were immediately arrested by small doses from time to time, the collapse diminished, and the stools made less frequent. The medicine is given in doses of 20 to 30 centigrammes in 90 grammes of infusion of chamomile. The child walks about in two days, and in six days health is restored. It is as antizymotic as phenic acid, but is not so irritating, and does not produce intoxication.—*Montpelier Medical.*

**Insomnia.**

Prof. Hoppe, in the Journal D'Hygiene, mentions a plan of treatment in insomnia which he has frequently found successful.

It consists simply in rapidly closing the eyelids, twenty or thirty times in succession, and thereby causing fatigue of the depressor palpebræ muscles; this will be followed in a few seconds by an irresistable desire for sleep. The professor recommends this treatment more especially in that form of insomnia which accompanies nervous diseases.—*Cincinnati Lancet and Clinic.*

**Laryngeal Phthisis.**

In laryngeal phthisis, says Dr. Bird (*Australian Medical Journal*), an inhalation with Siegle's spray, of filtered solution of hydrstin, with glycerine of borax and morphia, gives great temporary relief.—*Medical Record.*

**Morphia to the Moribund.**

Dr. Tubeldia recommends injections, hypodermic, to those suffering pain and are unconscious in the moribund condition. He said that morphine can for a few minutes reanimate a dying person, and enable him to express his last wishes. To a person perfectly unconscious, he injects chlorhydrate of morphine, one to two centigrammes, and if not conscious in four hours repeats the injection. The patient generally arouses, turns in his bed calls for wine and food, and a third injection brings a decided improvement.

**Hemorrhoids.**

Dr. Paul Laudowski, having tried all old remedies, astringents and hemostatics of every kind, concluded to try hot water—as hot as could be borne by the patient. After being in the bath some time, a small syringe is introduced in the rectum and the hot water gently introduced. The hemorrhage soon ceases, and does not return; the tumors collapse, and the bowel allows the finger to pass without pain.

The experiments are not sufficiently numerous to assert that all hemorrhoids will yield to the hot water treatment, but only to recommend the plan for farther experimentation.—*Journal de Therapeutique.*

**Hysteria.**

When called to treat a young girl with a hysterical attack there are three things which you had better do: 1. Institute at once firm pressure in the neighborhood of both ovaries. This is very apt to quiet the patient at once. 2. Administer an emetic. I have found that a woman who is well under the action of an emetic has not the opportunity to do anything else than be thoroughly nauseated. Give a full dose of ipecac with one grain of tartar emetic. 3. And this method of controlling the spasm will often act charmingly: take a good-sized lump of ice and press it right down on the nape of the neck. This produces quiet by its powerful impression upon the whole nervous system.—*Dr. Wm. Goodell in Clinical News.*

**Night Sweats.**

Da Costa considers ergotine the best remedy for the night sweats of phthisis—two grs. three or four times a day. It is less prompt than atropia, but it is free from any unpleasant after effects.

**Tænia.**

In the Antilles the cocoanut is a popular remedy in tænia, and its efficacy has been demonstrated by Dr. Martiali, chief of the medical service in Senegal. A five ounce cocoanut is opened, and the meat removed, scraped, and three hours after being administered, a dose of oil is given. In nine cases the success was complete where this remedy was taken.—*Revue de Therapeutique.*

**Kummerfeld's Lotion.**

This is intended an emollient application to the skin, particularly of the face. Its composition is the following:

Sublimed sulphur, 2 parts.
Glycerin, 2 parts.

Triturate them together until they are thoroughly mixed, then add

Glycerin, 10 parts.
Spirit of camphor (1:10), 4 parts.
Spirit of lavender, 10 parts.
Cologne, 10 parts.
Distilled water, 120 parts.

When wanted for use, it should be well shaken up.

**Contagiousness of Tubercular Matter.**

M. Toussaint has been making some interesting experiments with tuberculous matter from the human being.

He found that inert substances introduced into an organism, could cause a condition closely resembling acute tuberculosis, but that an animal inoculated with the product of this disease would not develop tuberculosis.

Further, when an animal is inocculated with the true tuberculosis matter, it will develop tuberculosis and the disease can be transmitted by inoculation an indefinite number of times When the poison is cultivated its virulence increases with each culture; that is, the tenth culture is more violent and rapid than the ninth and the fifteenth than the fourteenth. A rabbit inoculated with virus of the eighth culture, died in five months, with his lungs stopped up with tubercular product. A pig inoculated with vaccinia from a tuberculous cow, was killed and showed a general tuberculosis.—*La Medicale.*

**Atropia in Chordee.**

The following formula will be found of great value in Chordee. Sulphate of Atropia, four grains, Aqua-Rosa, two ounces, Misce: Signa. Saturate a little absorbent cotton with the solution, and, after retracting the prepuce, lay it behind the glans and push the prepuce over it. Repeat this procedure every three hours.

**Rectal Alimentation.**

From experiments made in Prof. Vulpian's laboratory, M. Catillon infers that in order to secure the full benefits of injected foods, they should first be transformed into peptones. For one enema he used: peptones of meat (the solution saturated at 19c) 40 grammes (about $1\frac{1}{2}$ ounce); water, 100 grammes (about $3\frac{1}{2}$ ounces); laudanum, a few drops, and bicarbonate of soda, 30 centigrammes ($4\frac{1}{2}$ grs.)—*D'Abeille Medicale.*

**Spermatorrhœa.**

Dr. Nowatschik, in two European journals, reports two cases (*Chicago Medical Review*) of spermatorrhœa cured by the use of atropia after all the usual remedies had been tried in vain. Iron quinine, cold applications to the genitals, and bromide of potassium were all used without results. In one of the cases he gave atropia by the mouth, and the patient completely recovered in five days. In the other case equally good results were obtained by injecting hypodermically into the perineum a one per cent. solution of atropia

**Headache.**

In severe nervous headache one grain of creosote taken in a cup of clear, strong coffee, is often speedily effectual.

In sick headache, heart burn and acid stomach, about forty drops of aromatic spirits of ammonia, taken in a wine glass of water, will frequently afford quick relief.

Persons who do a great deal of brain work are often the subjects of fierce headaches, due to hyperæmia of the nerve substance within the cranium. Tincture of pulsatilla in drop doses every two or four hours in a tablespoonful of water has been found of great service in such cases.

Oxalate of cerium, about three grains, taken dry on the tongue, sometimes acts like magic in relieving sick headache. The dose may be repeated as often as necessary.—*American Med. Journal.*

**Gonorrhœa.**

The injections are not very painful. They are used five times a day, the last injection being practiced just before retiring. They should be retained in the canal one or two minutes.

The following is the formula: Water, 150 grammes; Glycerine, 10 grammes; Bromide of Potassium, 6 grammes; Laudanum, 2 grammes. – *Journal de Therapeutique.*

---

**Uterine Hemorrhages.**

M. Cheron reports, with success, the treatment of hemorrhages by Dr. Ategre. From his investigations he considers capsicum particularly adapted to the utero-ovarian, respiratory and encephalic integuments. He has employed it many years in uterine hemorrhages with the best success, whether the hemorrhage proceed from fibrous tumors, fungous endometritis or epithelioma. The formulæ are the following:

Pulv. capsicum, 5 grammes, in 30 pills, one pill before each meal, or six in 24 hours.

The aqueous extract or tincture of capsicum with rum or gum julep also answers.

He has also employed capsicum to overcome, and with success, cephalalgia of the congestive form, so frequent among arthritics and in the hemorrhages from tubercles.—*Journal de Therapeutique.*

---

**Night Sweating.**

In the *Medical and Surgical Reporter* we find an article by Dr. F. P. Henry, of the Episcopal Hospital, Philadelphia, giving his experience in the use of picrotoxin, the alkaloid of the cocculus indicus, in night sweats. He has used it, not only in the sweating stage of consumption, but in other affections of the lungs in which this system became a prominent feature. Thus in a case of chronic pleurisy where there was absorption of the effused fluid, but where convalescence was unaccountably retarded and the skin bathed in perspiration, the picrotoxin was administered with marked success. He had used the remedy in nearly one hundred cases, mostly phthisis, in which prompt relief of the night sweats was obtained. Dr. William Murrell had previously obtained the same results. He gives one-eightieth of a grain at night, and repeats this amount during the day once or twice, if the case should prove obstinate. From these observations it would appear that picrotoxin has yielded better results in the night sweating of exhausting diseases than atropia, ergot or the mineral acids.

**Capsicum as an Emmenagogue.**

Capsicum is claimed by Cherou (Journal de Medicine et de Chirurgie, August, 1881), to be of great value as an emmenagogue, and to have similar properties to ergot. He gives the following formula:

℞

Tincture of capsicum, 45 grs.

Rum, 1 ℥.

Gum julep, 4 ℥.

Sig—Tablespoonful every two hours. He has also found it of value in pulmonary hemorrhage. *The Druggist.*

---

**Lister's Eucalyptus Gauze.**

This new dressing, devised by Lister, is prepared by impregnating gauze-muslin with the following mixture:

Oil of eucalyptus, 1 part.
Damar, 3 parts.
Paraffin, 3 parts.

The damar and paraffin are melted, the oil is added, and the mixture sprinkled or squirted over the muslin laid together in folds. It is then placed in an air-tight heating apparatus, compressed by weights, and exposed to dry heat. Occasionally it is taken out when cold, refolded, and again heated until it is uniform. The finished gauze contains 10 to 11 per cent. of the mixture; five square metres (about six yards of one-yard wide muslin) contain forty grains.

---

**Epilepsy.**

Dr. Hammond's experience has proved the following to be one of the best plans of treatment for epilepsy: Dissolve eight ounces of bromide of sodium in a quart of water. Of this, take a teaspoonful three times a day. After three months add one teaspoonful more to the afternoon dose also. At the expiration of a year, do the same with the morning dose, and continue this for a year or more thereafter. If no symptoms of the disease have meanwhile appeared, then gradually reduce the doses, and at the expiration of the third year stop. The attacks do not usually return after this course of treatment. Ordinarily, however, patients stop the medicine after a month or two, and in such cases the attacks almost invariably return. It is then almost impossible to bring these patients under the influence of the bromides again. The doses will have to be at least doubled, and this may so derange the system as to make it impossible to take the medicine longer.

**Epithelioma of the Neck of the Uterus.**

Dr. Cheron, referring to the Italian experiences with this practice, employs the nitrite of lead in ulcerating epithelioma of the uterine neck. After cleansing the surface with charpie moistened with glycerine, or washing out the canal with perchloride of iron solution if there is much oozing of blood, he applies to the ulcerated surface with an insufflator the following powder: Plumbi nitrat. purif. ʒ i. Lycopdii ʒij. The powder is kept in position by a suitable tampon. Under the action of this preparation the suppuration diminishes sensibly and the odor disappears. The hemorrhages are also suppressed. After twelve or fifteen of these applications, the engorgement of the cul-de-sac diminishes, and the general health is greatly improved.—*Med. News.*

**Dysentery.**

The treatment of dysentery by enemata of ice water is not new in this country, but the recent trial of it by Dr. Michaelov, of St. Petersburg, as quoted in the London *Medical Record*, is not without interest. He says it has not failed him in a single case for four years. It proved especially successful in the cases of his own children. A short description of two cases follows. Quinine, in the form of Botkin's cholera drops, was administered internally in all cases. (Compound tincture of quinine, Hoffman's anodyne spirit, of each half an ounce; hydrochlorate of quinine, one scruple; dilute hydrochloric acid, half a drachm; tincture of opium, half a drachm; peppermint oil, fifteen drops.) The diet consisted of bouillon, milk and eggs; the drink was cold water and red wine. The enemata were made in the following manner: Ice was crushed to paste; from this mass of ice two glasses were taken for grown up men for every enema; for children, according to age, beginning with half a glassful, and so on. Water was then added while the ice floated. It was then poured into a glass funnel connected with a long tube. This funnel, fixed on a stand, remained near the sick bed; in other words, the apparatus remained in action until the mass of ice melted and flowed slowly into the rectum, which process lasted about one or one and a half hours. The enemata were employed every two hours, then every three and four hours; finally twice or three times daily. The author succeeded in all cases in curing the disease in eight or ten days.—*Med. and Surg. Reporter.*

**Intestinal Worms.**

In a paper read before the Dearborn County Medical Society by Dr. C. Heaton, and published in the Cincinnati *Lancet and Clinic*, an account of an extraordinary collection of intestinal worms in a lady is detailed. The lady was pregnant, and frequently complained of nausea and irregular pains through the abdomen. To obtain relief from these symptoms she sent for the doctor. Having vomited a worm, the doctor suspected more, and gave her santonin, gr. iii; spir. terebinth, gtt. xx; hyd. chlor. mit., gr. xii; to be taken in the order mentioned, three hours apart. From the effects of these medicines she passed from the rectum seventy worms of the species ascaris lumbricoides, or common long round worm, each measuring from six to ten inches in length. This was on the 14th of February. He then gave her pink root, senna and worm-seed oil at different times, when she passed over eighty more by March 8th. She thus evacuated over 150 large worms in a period of 22 days. She then enjoyed good health and had a good delivery.

**Bronchial Affections of Pulmonary Phthisis.**

M. Melsens highly recommends (*Bull. de l' Academie Belge, et Paris Med.*,) the therapeutic employment of ammonia by inhalation in phthisis. Knowing that ammoniacal inhalations may be respired without danger, of which the proof is the perfect health of workmen who dig guano, and also knowing the good effects of the air of cow-houses in pulmonary phthisis, effects which are generally with justice attribute dto the emanations of carbonate of ammonia generated in these stables, M. Melsens conceived the idea that continuous but moderate respiration of this salt might be useful in other affections of the respiratory organs. He decided, after an attack of acute bronchitis, to make the experiment on himself. For that purpose, he wore outside his shirt a bag containing some pieces of carbonate of ammonia; after some time he was absolutely relieved from the affection; improvement set in from the first day. Several invalids who employed the same means obtained great benefit from it, even in cases of long-continued chronic bronchitis. Amongst others, a physician at Brussells, who had suffered for a long time from an obstinate cough, due to chronic bronchitis, with dilatation of the bronchi, complicated with emphysema, asthma, and sometimes with acute laryngitis. He used the bag of carbonate of ammonia, and found himself perfectly cured.—*London Med. Record.*

**Thymol in Diphtheria.**

The following mixture containing thymol has, we read in *Le Progres Medicale*, proven very efficacious in Dr. Warren's hands:

℞

Thymol, gr. ivss-viss.

Potass. chlorat., ʒ iiss.

Quiniæ bisulph., ʒ ss- ʒ j.

Sp. vini gall., ℥ viiiss.

Glycerinæ, ℥ ij.

M. For children from two to five years of age, a dessert-spoonful may be given every hour or every second hour.

The mixture should be given, if possible undiluted, in order to obtain its excitant, almost irritant action on the buccal parietes.

This mixture may also be employed as a prophylactic agent against diphtheria and malaria. By the addition of a few drops of tincture of iron to each dose it acts as a tonic and may be employed with success in typhoid fever when diarrhœa is present.—*Med. and Surg. Reporter.*

---

**Whooping Cough.**

Bromide of ammonium vaunted in the treatment of whooping-cough by Harley, Gibb, and Ritchie, has lately been tried again at the Dresden Clinique for Diseases of Children, with the best results (LaFrance Medicale). Korman has convinced himself that from the first doses the frequency and intensity of the fits of coughing diminish. The bromide is administered as follows: Every two hours 15 centigrammes for children under one year of age ; 25 to 50 centigrammes for older children, suspended in coffee or in a draught sweetened with liquorice. The medicine should be taken after coughing to avoid its being vomited. Even very young children bear relatively large doses of the remedy. At most there is observed sometimes apathy and somnolence, which disappear in a few hours on the remedy being suspended. The existence of gastric catarrh is a contra-indication for the employment of bromide of ammonium.—*Medical Press and Circular.*

---

**Frontal Headache.**

The London *Medical Times and Gazette* says: Dr. Haley states, in the *Australian Medical Journal* for August, that for some time past he has found minimum doses of iodide of potassium of great service in frontal headache. A heavy dull headache, situated over the brow and accompanied by languor, chilliness, and a feeling of discomfort, with distaste for food, which sometimes approaches to nausea, can be completely removed by a two-grain dose, dissolved in half a wine glass of water, and this quietly sipped, the whole quantity being taken in about ten minutes. In many cases the effect of these small doses has been simply wonderful. A person who, a quarter of an hour before, was feeling most miserable and refused all food, wishing only for quietness, would now take a good meal and resume his wonted cheerfulness. The rapidity with which the iodide acts in these cases constitutes its great advantage.—*Medical and Surgical Reporter.*

---

**Chloral Hydrate and Diabetis.**

According to the Glasgow *Medical Journal* (*Medical Summary*), Dr. Eckhard injected a quantity of chloral under the skin of a dog, and then penetrated the fourth ventricle of the brain; no sugar appeared in the urine. In a second animal, glucosuria was first produced by penetrating the floor of the fourth ventricle; then chloral was injected as before, and the sugar disappeared. He was able to produce glucosuria by stimulating the proximal end of the vagus nerve, where section of this nerve was made in the neck; but the experiment failed after chloralibation. No sugar appeared in the urine of a dog, when made to breathe carbon monoxid, when chloral (5 grains) had previously been administrated. The marked influence of chloral over the excreation of sugar by the kidneys was turned to good account in the treatment of two cases of diabetis mellitis. The exhibition of chloral in these cases was accompanied by a marked diminution in the quantity of urine eliminated, and in the amount of sugar it contained.

---

**Chronic Rheumatism.**

The name rheumatism is applied to a great number of affections appearing alike but differing one from another—by one person being considered an inflammation, by others a pyrexia—and having different treatment—salts, alkalies, sulphate quinine, salicylate of soda, etc., etc., the latter of which is excellent in acute rheumatism and often in sciataca.

The word rheumatism as employed expresses different morbid states, as scrofula, syphilis, etc., differing in appearances. In our knowledge of the disease it is not possible to affirm that all the manifestations of rheumatism depend on the same cause. Cold is often the occasional cause and often the efficient.

In the uric acid diathesis, the alkaline bicarbonate mineral waters are the best, as Vichy, Vals, Curset.

Hydropathy is sometimes a powerful adjuvant in different forms of rheumatism, and Six in Savoy is the best therapeutical station in Europe.—*Dr. Coiquard, in Journal de Therapeutique.*

**Amenorrhea.**

A large number of remedies have been credited with emmenagogue properties, many of them being inert, and some of them simply irritant poisons whose employment has frequently resulted fatally, especially when used with criminal intent, as abortifacients. Strychnia affords excellent results in many instances. A favorite with me is the following :

℞

Strychniæ sulph, gr. j.

Dextro-Quininæ, ʒ j.

Ferrum per hydrogen, } aa ʒ ij.

Asafeitidæ pulv., }

Ext. qussiæ, q. s. M.

In pil. No. 60 div. Sig.—One four times daily.

I usually add at bed time ten drops of Squibb's fluid ext. ergot in water; and a forcible jet of cold water along the spine every morning on rising for a few minutes, with brisk friction of the abdomen, succeeded admirably in many cases. Exercise in the open air, equestrianism particularly, with attention to a normal action of the skin, kidneys, and bowels is essential.—*Review Med. & Pharmacy.*

**Transfusion.**

Dr. Erich spoke of transfusion in cases of collapse from postpartum hemorrhage. In one case he had tried Aveling's instrument, using the arm of the patient's son to get the blood, but could not get the blood to flow, because clots formed in the instrument. He thought defibrinated blood should be employed. He preferred auto-transfusion. About ℥ vj of blood is all that can be safely introduced by transfusion, and this amount can generally be obtained from the lower extremities by squeezing and bandaging them and elevating the feet of the bedstead. Electricity is one of the most efficient agents to excite contractions.

In an experiment upon a dog he bled the animal "within an inch of his life," then injected the defibrinated blood back into his veins, and the animal revived.

Dr. Reiche had witnessed the use of the transfusion apparatus once only, and then the results did not impress him favorably. The patient had a uterine fibroid, and bled frightfully at each monthly period. She lingered on, however, recovering each time until the transfusion ; although conscious before this was practiced, she was not so afterwards, and her death occurred in two days.—*Va. Med. Monthly.*

**Facial Neuralgia.**

I have had a good deal of experience in the use of gelsemium, both in my own person and that of patients. I have found it a reliable and safe drug in suitable cases, such being cases of neuralgia of the dental nerves due to carious teeth. In my own personal case, I always have recourse to it, and with success, as soon as ever I feel a twinge in a decayed tooth ; and I have given it to patients under similar circumstances, with like favorable results when taken soon enough. In cases of facial neuralgia, however, involving other parts of the face, as the forehead and the temples, I have not had good results from it, and have latterly always limited its employment to cases where the neuralgia was apparently due to the condition of the teeth.

I usually prescribe the tincture in doses of 10 or 15 minims, repeatedly every half hour or hour until the pain is relieved. I have never seen or experienced any deleterious effects, and, in my own case, have taken it at all times of the day and night, and in all positions.—W. D. Hemming, F. R. C. S. Ed. in *The Brit. Med. Jour.*

**Frequently Recurring Erysipelas of the Face.**

This affection is very annoying to the patient, for, in spite of every precaution, it will recur again and again. If any cause can be discovered, such as bad drainage, it should at once be remedied ; but, whatever other hygienic of medical treatment be employed, some local application is generally necessary. All these applications are either disfiguring or disagreeable, or totally inefficient. For many years, my father and I have used with entire success a strong solution of tannin (four to eight grains to the drachm of spirits of wine and water.) This application, which is not disagreeable to the patient, should be painted over the parts affected with a soft brush every two or three hours, and allowed to dry, the patient being careful to keep the face from the fire. If there be a tendency to frequently recurring erysipelas, it is well to keep the tannin at hand, as it will always arrest a threatened attack.—*James Braithwaite, M. D., Leeds, in British Med. Jour.*

**Disinfection of Urine.**

Disinfectant properties are claimed for various substances in connection with the urine. Among these, nitrite of amyl has been tested. It has been used with this view for chronic catarrh of the bladder, by injection, with favorable results, it is said. It has also been made use of for the preservation of the urine.

It may not be generally known that hydrate of chloral has similar properties, of which I have a marked proof. In August, 1877, Dr. C. H. Porter, of this city, gave me a specimen of urine, four ounces, containing five grains of chloral to the ounce. No special care has been taken to facilitate its preservation. It has been simply without sealing, corked, and has several times been opened, though standing most of the time quietly in a dark cupboard. It is now perfectly transparent, is of a clear amber color, and has a fresh, urinous, slightly balsamic odor. A slight, semi floculent deposit covers the bottom of the vial. The chemical analysis is identical with the notes made when it was recent: specific gravity, 1015, acid, albumen, one-fourth. The preservation of the albumen is a marked test. On microscopic examination, the epithelial cells are as perfect as in recent urine. No casts were noted (the case was one of Bright's disease, long since terminating fatally), and none are now seen. A few octahedral crystals, identical with calcium oxalate, except that they do not show the shining edges, are now seen; no note is made of them in the fresh examination, but they may be oxalate of lime, not then, possibly, having deposited; for, being light of weight, they fall slowly, and sufficient time may not have been given for them to deposit. The specimen seems to be quite the same as when freshly voided, and is manifestly instructive and of considerable interest.—*F. C. Curtis, M. D., Albany, in Ther. Gazette.*

---

**Diphtheria.**

Dr. Weise (*Berlin, Klin. Wochenschrift*) advocates the adoption of the following measures in the treatment of diphtheria: He prescribes a solution of salicylic acid, consisting of one part of salicylic acid and twenty-five parts each of rectified spirit and glycerine. This, although a strong solution, is quite free from danger. The spirit, he explains, is used because, in former cases, benefit was derived from the local application of brandy to the throat; and the glycerine is added to obtain a less irritating solution If the patient can gargle, the author employs a 1 in 300 salicylic acid gargle. Should the attack be very severe, he prefers a solution of benzoate of soda, 1 in 40, or even stronger. Coincidently with this treatment, he gives Hungarian wines. He pays particular attention to maintaining the patient's strength, and orders nourishment to be taken constantly, in the form in which it can be most readily assimilated. He speaks highly of Rosenthal's extract of meat mixed with yolk of egg, but gives solid food if it can be borne. His directions are somewhat as follows: At one o'clock, paint or gargle the throat with the salicylic acid solution; at half-past one, give a teaspoonful of Hungarian wine; at two, the benzoate of soda; at half-past three, a teaspoonful of Hungarian wine; and, during the next half hour, more gargling with salicylic acid; the treatment being continued night and day without intermission, except that at night the intervals may be an hour instead of half an hour. If possible, he employs a special apparatus of his own—a combination of tongue-depressor and spray. This, by reaching the back of the tongue, admits of a thorough examination with the simultaneous application of the antiseptic. In a few seconds, the whole throat can be washed out with the salicylic acid solution; and this is very necessary in preventing the spread of the disease. Moreover, by this method, bleeding may be speedily arrested. Sometimes he employs a 5 per cent. solution of carbolic acid as a spray.—*British Med. Journal.*

---

**Bright's Disease.**

Since we know not at present any drug that possesses therapeutic value to any marked extent in this terrible and fatal disease, and since it is daily making sad havoc among human beings, and principally among that class who, by reason of their valuable public labors, are particularly necessary to the welfare of the world; therefore, it becomes a medical question of paramount interest that we should discover some potent method of combatting this very prevalent disease. Some years since Carel first called attention to the treatment of Bright's disease by the use of a milk diet, and since then Duncan, as well as many other prominent phyicians, have written on this subject. We have ourselves seen some remarkable results follow this treatment, while Dr. S. Weir Mitchell, of our city, is now quite an enthusiast on this subject. This method of treating a formidable disease has received sufficient distinguished endorsement to recommend it seriously to our notice. We would, therefore, ask all physicians who read this article to try this method of treatment, and to furnish us with their experience, which we will publish. The milk is used thoroughly skimmed, and entirely freed from butter. To procure the best results, it has been advised that the patient shall restrict himself absolutely to milk, and continue the treatment for a long time. If it disagrees with the stomach (as it will in some cases), Dr. Mitchell advises that the patient be put to bed, and the treatment commenced with tablespoonful doses, to which lime water is added, until the stomach tolerates the milk, when from 8 to 10 pints daily should be taken, and absolutely nothing else. The sanction of such a distinguished physician as Dr. Mitchell forces us to seriously consider the merits of this treatment, and we trust to receive the experience of all readers of this journal who may have cases of Bright's disease to treat.—*Med. and Surg. Reporter.*

**Can a Threatened Attack of Diphtherta be Averted ?**

Dr. Alfred Sheen, Surgeon to the Cardiff Infirmary, writes to the editor of the London *Lancet* on this important subject. His question is based on this fact : He was called to see a young woman who had nursed her brother through a fatal case of diphtheria. Four days after his death she was seized with general malaise, shivering and thirst. Pulse, 144. Temp. 103.4°. She did not complain of her throat, which, on examination, presented a normal appearance. She was given twenty-five grains of ipecacuanha powder at once, and one ♏ of the tincture of aconite every quarter of an hour, for the first hour, and every hour afterward. The next day her pulse was 94 and temp. 99.8°. On the next day her *throat was painful and congested,* but no patches. She was ordered :

℞

Liq. ferri perchlor, f ʒ iv.

Potass. chlorate, ʒ ij.

Glycerinæ, f ʒ ij.

Aquæ, f ℥ vj.

M. Sig.—f ℥ ss every four hours, in water.

Two days afterward pulse was 76 and temp. 98°. Congestion gone and the girl said she was quite well. Dr. Sheen then asks : "Was this woman, when I first saw her, suffering merely from a sharp attack of febricula, brought on by fatigue, anxiety about her brother, and subsequent exposure at the funeral, or was she in the early stage of diphtheria ?" He adds that, "As a rule, specific febrile diseases are not averted by treatment, but may that not be because we do not get hold of the disease early enough." (?) In view of the fatal nature of this disease, and the uncertainty surrounding its pathology and therapeutics (so great that a distinguished French lady has offered a reward of five thousand dollars to any one who may discover a preventive), this query is worthy of attention and investigation, and we will be glad to hear from our readers on the subject.—*Med. and Surg. Reporter.*

**Acute Tonsilitis.**

To conclude our exercises this morning, I will show you a case, not of very great interest in itself, but one that is frequently encountered in private practice, and therefore important for you to see.

This man is suffering with violent acute tonsilitis ; the tonsils, as you can see, are still inflamed; they were enormously swollen. He had, when he came in, eight days ago, considerable fever ; the temperature at one time was 103½°, but it did not long remain at this height. The attack began with a chill, soon followed by fever, mucous expectoration, sore throat, but nothing like a diphtheric deposit upon the surface. Both tonsils were swollen ; he had high fever. It was a case of more than average severity, and in such cases the attack usually terminates with suppuration of the tonsils. It is rare that treatment will succeed in making the inflammation subside without it.

We gave him ten grains of quinine daily, at first in a single morning dose ; afterwards in divided doses. We allowed him to suck ice freely; and also, bearing in mind our recent case of parotid swelling, we applied the ice in bags to the outside of the throat, assiduously. This was carried out very effectually; for in place of the profuse suppuration which usually takes place in such cases in the tonsils, it has only been superficial and very slight, and has affected only one tonsil. Therefore we have reason to be pleased with the effects of the ice and quinine treatment in this case. Otherwise nothing locally was done ; he used a little water as a gargle but no astringents; we relied solely upon the ice which he sucked, and had applied to the angles of the jaw.

Now, gentlemen, this is a frequent disease and is very painful; if suppuration occur it is prolonged. I have called your attention to it solely with regard to its therapeutics; it presents no difficulty in diagnosis, though obscure in its pathology. In severe cases I believe that the treatment followed in this patient promises very satisfactory results.—*Clinic of Dr. J. M. Da Costa—College and Clinical Recoxd.*

